# Preliminary study on radiosensitivity to carbon ions in human breast cancer

**DOI:** 10.1093/jrr/rraa017

**Published:** 2020-04-02

**Authors:** Qiuning Zhang, Yarong Kong, Zhen Yang, Yang Liu, Ruifeng Liu, Yichao Geng, Hongtao Luo, Hong Zhang, Hongyan Li, Shuangwu Feng, Xiaohu Wang

**Affiliations:** 1 Institute of Modern Physics, Chinese Academy of Sciences, Lanzhou 730000, China; 2 The Life Sciences College of Lanzhou University, Lanzhou 730000, China; 3 Basic Medical College of Lanzhou University, Lanzhou 730000, China; 4 The First Clinical Medical College of Lanzhou University, Lanzhou 730000, China; 5 Gansu Provincial Cancer Hospital, Lanzhou 730050, China; 6 Lanzhou Heavy Ion Hospital, Lanzhou 730030, China

**Keywords:** carbon ions, breast cancer, MDA-MB-231, MCF-7, Akt/mTOR/p70S6K pathway, radiosensitivity

## Abstract

The aim of the study was to investigate the various effects of high linear energy transfer (LET) carbon ion (^12^C^6+^) and low LET X-ray radiation on MDA-MB-231 and MCF-7 human breast cancer cells and to explore the underlying mechanisms of radiation sensitivity. Cell proliferation, cell colony formation, cell cycle distribution, cell apoptosis and protein expression levels [double-strand break marker γ-H2AX, cell cycle-related protein cyclin B1, apoptosis-related proteins Bax and Bcl-2, and the Akt/mammalian target of rapamycin (mTOR)/ribosomal protein S6 kinase B1 (p70S6K) pathway] were detected after irradiation with carbon ions or X-rays at doses of 0, 2, 4 and 8 Gy. Our results showed that the inhibition of cell proliferation and cell colony formation and the induction of G_2_/M phase arrest, DNA lesions and cell apoptosis/necrosis elicited by carbon ion irradiation were more potent than the effects elicited by X-ray radiation at the same dose. Simultaneously, compared with X-ray radiation, carbon ion radiation induced a marked increase in Bax and prominent decreases in cyclin B1 and Bcl-2 in a dose-dependent manner. Furthermore, the Akt/mTOR/p70S6K pathway was significantly inhibited by carbon ion radiation in both breast cancer cell lines. These results indicate that carbon ion radiation kills MDA-MB-231 and MCF-7 breast cancer cells more effectively than X-ray radiation, which might result from the inhibition of the Akt/mTOR/p70S6K pathway.

## INTRODUCTION

Breast cancer is the most common type of cancer among women in Asia, accounting for 23% of all malignant tumours [1]. There are 1.3 million people worldwide diagnosed with breast cancer every year, and ~400 000 people die of breast cancer [2, 3]. In recent years, the incidence of breast cancer has been increasing at a rate of 2–3% per year, and it has ranked first in female malignant tumours in China. Breast cancer is divided into different subgroups based on whether it expresses oestrogen receptor (ER), progesterone receptor (PR) or human epidermal growth factor receptor 2 (HER2) [4]. Radiotherapy, as one method of standard breast cancer therapy, is indispensable in the comprehensive treatment of breast cancer and helps to reduce the frequency of distant metastasis and the mortality rate by blocking the local recurrence of breast cancer. Some research has confirmed that the radiosensitivity of the ER-negative breast cancer cell line MDA-MB-231 is significantly lower than that of the ER-positive breast cancer cell line MCF-7 [5, 6].

With traditional radiotherapy, such as X-ray irradiation, it is difficult to achieve the desired efficacy because of radiation resistance in cancer cells [7]. High-linear energy transfer (LET) radiation (normally >10 keV/μm) demonstrates an increase in energy deposition with a penetration depth up to a sharp maximum at the end of its range, known as the Bragg peak, which endows high-LET heavy-ion beams with an excellent dose distribution, allowing precise localization of a sufficient dose in the target lesion area while minimizing the damage to the surrounding normal tissues [8]. Moreover, charged particles have a higher LET, which ensures a higher relative biological effectiveness than conventional X-rays [9–11]. Many reports have certified that heavy-ion beams have advantages over conventional radiation in various kinds of cancer cells, such as HeLa cells, malignant melanoma cells, glioma cells, colorectal cancer cells, lung cancer cells and breast cancer cells, through different molecular mechanisms [12–16]. Recently, carbon ions have attracted much interest and become an increasingly available option for the treatment of various malignancies [17, 18], and they show higher local control and survival rates than other radiotherapy modalities for various types of tumour [19, 20]. Because of its higher LET and better dose distribution, heavy-ion irradiation can mainly induce more serious damage, such as oxidative stress and carcinogenesis, and more DNA damage, especially DNA double-strand breaks (DSBs), than X-ray irradiation. DSBs are the most lethal, as an accumulation of misrepaired or unrepaired DSBs can lead to a massive loss of genetic information and cell death in mammalian cells and tissues [20]. The phosphatidylinositol 3-kinase (PI3K)/Akt/mammalian target of rapamycin (mTOR) pathway plays an important role in regulating cell growth, survival, adhesion and migration in response to growth factors and nutrient availability [21]. This pathway is also known to be overstimulated in cancer cells and plays an important role in cancer progression, metastasis, and in tumours resistant to treatments such as chemotherapy, radiation and hormone therapy [22]. mTOR is a key node of the PI3K/Akt/mTOR pathway, belongs to the phosphatidylinositol-3-OH kinase-associated kinase family and is located on the surface of lysosomes. As an important serine/threonine kinase that maintains homeostasis, mTOR plays an important regulatory role in protein translation, glucose and lipid metabolism, cell survival and autophagy [23]. Akt is one of the main predictors of the radiation response. As the upstream protein of mTOR, activated Akt regulates the repair of DNA DSBs induced by ionizing radiation and thus affects the radiation resistance of solid tumours [24]. Ribosomal protein S6 kinase B1 (P70S6K) is a downstream protein of mTOR that responds to intracellular ATP levels and nutritional status by phosphorylating the serine-2448 site of mTOR [25]. Recent findings suggest that dual targeting of mTOR has the ability to overcome resistance to ionizing radiation and improve the anticancer efficacy of treatment in various cancer cell types in *in vitro* and *in vivo* models of cancer [26].

Recent findings suggested that carbon ion irradiation downregulates the Akt/mTOR pathway to induce autophagy in SHG44 and HeLa cells and suppress cell growth in human squamous cells [14, 27]. To confirm whether the AKT/mTOR/p70S6K signalling pathway is involved in the lethal effects caused by carbon ion and X-ray irradiation, the radioresistant triple-negative breast cancer cell line MDA-MB-231 and the more sensitive MCF-7 (ER^+^/PR^−^/HER2^−^) cancer cell line were used to detect cell proliferation, cell colony formation, cell cycle distribution, DNA damage, cell apoptosis and Akt/mTOR/p70S6K signalling pathway activity in both cell lines at 48 h after exposure to two different types of radiation. Our results demonstrated that carbon ion irradiation inhibited cell proliferation and cell colony formation, induced G_2_/M cell cycle arrest, DNA lesions and cell apoptosis in MDA-MB-231 and MCF-7 cells, and the activation levels of Akt/mTOR/p70S6K were more efficiently decreased by carbon ion irradiation than by X-ray irradiation. Thus, we speculated that the Akt/mTOR/p70S6K signalling pathway may take part in the regulation of the radiation sensitivity to the carbon ions in MDA-MB-231 and MCF-7 cells.

## MATERIALS AND METHODS

### Chemicals and reagents

Phosphate-buffered saline (PBS), 0.25% trypsin, Dulbecco’s modified Eagle’s medium (DMEM)–high glucose medium, penicillin–streptomycin and foetal bovine serum (FBS) were purchased from HyClone (Logan, UTh, USA). The Cell Counting Kit-8 (CCK-8) was from BestBio (Shanghai, China). Fluorescein isothiocyanate (FITC)-conjugated annexin V/propidium iodide (PI) was purchased from BD Biosciences (USA). Radioimmunoprecipitation (RIPA) lysate, protein quantification kit, 4× protein loading buffer and enhanced chemiluminescence (ECL) hypersensitive chemiluminescence were from Solarbio (Beijing, China). The cell cycle kit was purchased from Sigma-Aldrich (St. Louis, MO, USA). Rabbit monoclonal antibodies (rabbit anti-human) against p-Akt (Ser473), Akt, p-mTOR (Ser2448), mTOR, p-p70S6K (Thr389), p70S6K, Bcl-2, Bax, cyclin B1, γ-H2AX and β-actin were purchased from Abcam (Cambridge Science Park, Cambridge, UK). Secondary horseradish peroxidase-labelled goat anti-rabbit IgG was purchased from Zhongshan Jinqiao (Beijing, China).

### Cell culture

Human breast cancer cell lines MDA-MB-231 and MCF-7 were obtained from Wuhan Boster Biotech and cultured in DMEM–high glucose medium supplemented with heat-inactivated 10% FBS, 100 U/mL penicillin and 100 μg/mL streptomycin in a humidified incubator at 37°C and 5% CO_2_.

### Irradiation

#### Carbon ions

Cells were trypsinized and seeded in a 35-mm culture dish for 24 h in culture medium when their growth was in the log phase. The irradiation was performed with carbon ion beams of 80.55 MeV/μ in the heavy-ion therapy terminal of the Heavy Ion Research Facility in Lanzhou (HIRFL) at the Institute of Modern Physics (IMP), Chinese Academy of Sciences. The dose-averaged LET of the carbon ion beams used on the cell samples was ~50 keV/μ, and the dose rate was approximately 2 Gy/min.

#### X-rays

Cells were plated in T25 flasks and cultured for 24 h before irradiation and subsequently irradiated with X-rays, which were generated using an X-rays instrument (Faxitron RX-650, Faxitron Bioptics, LLC, Tucson, AZ, USA) operated at 60 kVp. The dose rate was ~2 Gy/min. All irradiation treatments were carried out at room temperature.

### Cell proliferation assay

The cell growth of MDA-MB-231 and MCF-7 human breast cancer cells was determined by CCK-8 assay. Cells were seeded in 96-well culture plates at a density of 4000 cells in a final volume of 200 μL/well, with 6 parallel wells for each set of experiments, after different doses of carbon ion and X-ray irradiation (0, 2, 4 and 8 Gy). Cells were cultured normally for 24, 48 and 72 h, respectively, and then supplemented with 10 μL of CCK-8 reagent per well and cultured for an additional 2 h in the incubator. The optical density (OD) value of each well was determined by the microplate reader (Bio-rad 680, USA) at a wavelength of 450 nm.

### Cell colony forming assay

MDA-MB-231 and MCF-7 human breast cancer cells, seeded in 6-well plates at 2000 cells per well with 3 parallel wells per group, were incubated at 37°C overnight. Fourteen days after irradiation with X-rays or carbon ions at doses of 0, 2, 4 and 8 Gy colonies were fixed with methanol and stained with 1% Giemsa solution (Solarbio, Beijing, China). Colonies containing at least 50 cells were scored.

### Cell cycle distribution analysis

MDA-MB-231 and MCF-7 human breast cancer cell lines were plated in a 35-mm culture dish for carbon ion irradiation and seeded in T25 flasks for X-ray irradiation. They were maintained in fresh culture medium. Cells were cultured 48 h after carbon ion and X-ray irradiation (0, 2, 4 and 8 Gy), digested with 0.25% trypsin, harvested (including the cells from the supernatant), washed twice with precooled PBS, fixed in ice-cold 75% ethanol (100% ethanol dissolved in PBS) and stored at −20°C overnight. The following day, the cells were centrifuged, the 75% ethanol was discarded and the pellet was hydrated with sufficient PBS for 15 min; the sample was centrifuged again, PBS was discarded, and then the cells were stained with 300 μL of PI (1 μg/mL, Sigma) in the presence of 1% RNase A for 30 min. The cell cycle distribution was detected by a flow cytometer (BD, FACSCalibur, USA) at an excitation wavelength of 488 nm and an emission wavelength of 630 nm. A total of 20 000 single cells per sample were collected, and the ratio of the cells in the G_0_/G_1_, S and G_2_/M phases was analysed by ModFit 5.0.

### Cell apoptosis analysis

Cell apoptosis was examined by a double-staining method using a FITC-labelled annexin V/PI apoptosis detection kit. Briefly, cells were cultured normally for 48 h after X-ray and carbon ion irradiation (0, 2, 4 and 8 Gy), harvested (including the cells from the supernatant), washed twice with precooled PBS and resuspended in 300 μL of binding buffer. The two compensation groups were stained only with 5 μL of FITC-conjugated annexin V or 5 μL of PI. The control group and irradiation groups were all stained with 5 μL of FITC-conjugated annexin V and 5 μL of PI dyes per sample and then incubated away from light for 15 min at room temperature. The externalization of phosphatidylserine and the permeability to PI were evaluated using a flow cytometer (Amnis FlowSight, Seattle, WA, USA), and 10 000 single cells per sample were collected. Cells in early stages of apoptosis were positively stained with annexin V, whereas cells in late apoptosis were positively stained with both annexin V and PI. The cell apoptotic rate was analysed by IDEAS Application v6.0, and the apoptosis rate of both the control group and irradiation group was standardly done by the compensation groups.

### Western blot analysis

After X-ray and carbon ion irradiation, the cells were washed twice with precooled PBS. The RIPA lysate containing protease inhibitor was added to lyse the cells for 20 min on ice, and the supernatant was then collected. The BCA (Bicinchoninic acid) kit was used to quantify and adjust the total protein to 40 μg per sample. Quantified protein supernatant was supplemented with 4× protein loading buffer proportionally, boiled for 10 min to denature the protein, and stored at −80°C. An equal amount of each total protein sample was separated by 10, 15 or 6% sodium dodecyl sulfate polyacrylamide gel according to the molecular weight of the target protein and then transferred to a polyvinylidene difluoride membrane. Membranes were blocked with Tris-buffered saline containing 0.5% Tween-20 and 5% dried skimmed milk powder for 2 h at room temperature, incubated overnight with the primary antibodies at 4°C and subsequently incubated with the appropriate secondary antibodies for 2 h at room temperature. Signals were detected using an ECL kit. Fluorescence intensities were measured by FluorChemR Imaging Systems (AlphaInnotech, San Leandro, CA, USA). Quantification of protein signals was performed with Gel-Pro Analyzer software. The primary antibodies used in the western blot analysis included human p-Akt, Akt, p-mTOR, mTOR, p-p70S6K, p70S6K, Bcl-2, Bax, cyclin B1, γ-H2AX and β-actin.

### Statistical analysis

Data are presented as the mean ± standard deviation (SD). Comparisons of multiple groups were evaluated by one-way analysis of variance (ANOVA) followed by Tukey’s multiple comparison procedure. *P* < 0.05 was considered statistically significant.

## RESULTS

### Carbon ion irradiation decreases the survival of MDA-MB-231 and MCF-7 human breast cancer cell lines more effectively than X-ray irradiation

**Fig. 1.**
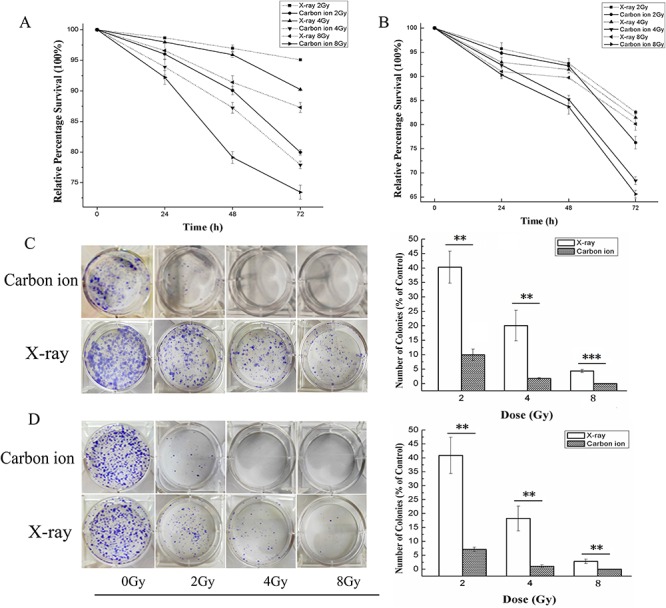
The effects of X-ray or carbon ion irradiation on the proliferation and colony formation of MDA-MB-231 cells (**A**) and MCF-7 cells (**B**) determined by 48-h CCK-8 assay. The cells were irradiated by X-rays or carbon ions at doses of 0, 2, 4 and 8 Gy, and then the OD values of all samples were determined under the same conditions. (**C**) and (**D**) show the colony formation results and the relative percentage of colonies of MDA-MB-231 (C) and MCF-7 (D) cells after irradiation with X-rays or carbon ions. The values are presented as a percentage of the control treatment (0 Gy). The results are the mean ± SD of three different experiments. ^**^P < 0.01 and ^***^P < 0.001 by one-way ANOVA.

The dose-dependent inhibitory effects of X-ray or carbon ion irradiation on the growth of MDA-MB-231 and MCF-7 cells are shown in Figs 1A and B. In MDA-MB-231 cells (Fig. 1A), at 24 h post-irradiation, the relative survival rates with irradiation at doses of 2, 4 and 8 Gy compared with 0 Gy (100%) were 98.67 ± 0.19, 97.97 ± 0.07 and 96.59% ± 1.46 for X-ray irradiation and 96.02 ± 0.16, 93.92 ± 1.28 and 92.22% ± 1.15 for carbon ion irradiation, respectively. At 48 h post-irradiation, the relative survival rates were 96.96 ± 1.54, 95.90 ± 1.44 and 91.41% ± 1.08 for X-ray irradiation and 90.05 ± 1.69, 87.24 ± 0.90 and 79.11% ± 0.99 for carbon ion irradiation. At 72 h post-irradiation, the relative survival rates were 95.08 ± 0.14, 90.22 ± 0.22 and 87.30% ± 0.80 for X-ray irradiation and 79.96 ± 0.44, 77.91 ± 0.63 and 73.44% ± 1.14 for carbon ion irradiation. In MCF-7 cells (Fig. 1B), the relative survival rates at 24 h after irradiation were 95.71 ± 1.23, 92.91 ± 1.01 and 90.99% ± 1.06 for X-ray irradiation and 94.80 ± 0.35, 92.33 ± 0.12 and 90.28% ± 0.03 for carbon ion irradiation. The relative survival rates at 48 h after irradiation were 95.71 ± 0.97, 91.38 ± 1.69 and 89.72% ± 1.11 for X-ray irradiation and 92.14 ± 1.16, 85.22 ± 0.79 and 83.68% ± 1.52 for carbon ion irradiation. The relative survival rates at 72 h after irradiation were 82.53 ± 0.40, 81.44 ± 1.05 and 80.11% ± 1.27 for X-ray irradiation and 76.27 ± 1.30, 68.41 ± 0.79 and 65.61% ± 0.93 for carbon ion irradiation. These results indicated that the relative survival rate decreased in a time-dependent and dose-dependent manner after exposure to X-rays or carbon ions. In addition, carbon ion irradiation showed significant superiority over X-ray irradiation in inhibiting the proliferation of MDA-MB-231 and MCF-7 human breast cancer cells at the same dose, especially for MDA-MB-231 cells.

### Carbon ion irradiation inhibited the surviving fraction of MDA-MB-231 and MCF-7 human breast cancer cell lines more effectively than X-ray irradiation

To compare the different effects of X-ray and carbon ion irradiation on MDA-MB-231 and MCF-7 human breast cancer cells, a colony forming assay was performed after exposure to the two different radiation types at doses of 0, 2, 4 and 8 Gy, and the results are shown in Figs 1C and D. In MDA-MB-231 cells, after X-ray irradiation, the relative percentages of colonies after treatment with 2, 4 and 8 Gy compared with 0 Gy were 40.31 ± 5.55, 20.07 ± 5.29 and 4.35% ± 0.58, respectively, while they were 9.97 ± 1.95, 1.77 ± 0.28 and 0%, respectively, after carbon ion irradiation. In MCF-7 cells, the relative percentages of colonies were 40.88 ± 6.52, 18.23 ± 4.45 and 2.83% ± 0.81 after X-ray irradiation, while they were 7.14 ± 0.80, 0.001 ± 0.003 and 0% after carbon ion irradiation. These results indicated that the survival of both human breast cancer cell lines was decreased in a dose-dependent manner after exposure to X-rays or carbon ions. Furthermore, the relative percentage of colonies formed in response to carbon ion irradiation was lower than that in response to X-ray irradiation at the same dose in MDA-MB-231 and MCF-7 cells (*P* < 0.001 by one-way ANOVA).

### Carbon ion irradiation induced G_2_/M phase arrest more effectively than X-ray irradiation in MDA-MB-231 and MCF-7 human breast cancer cell lines

**Fig. 2.**
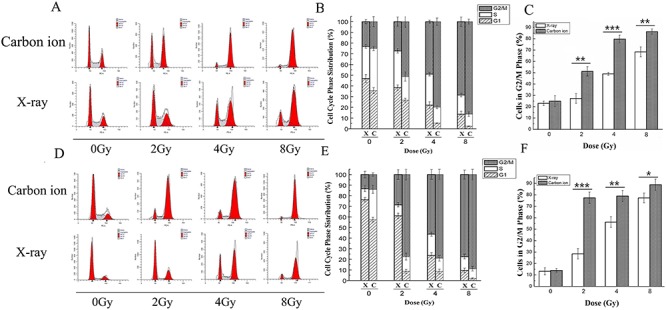
The different effects of X-ray and carbon ion irradiation on the cell cycle phase distribution in MDA-MB-231 and MCF-7 cells. The cells were irradiated by X-rays or carbon ions at doses of 0, 2, 4 and 8 Gy, and then the cell cycle phase distribution was determined by flow cytometry 48 h after irradiation. The DNA fluorescence histograms show the distribution of specific cell populations in the G_1_, S and G_2_/M phases in MDA-MB-231 (**A**) and MCF-7 (**D**) cells. The stacked plot shows the percentage of cells in the G_1_, S and G_2_/M phases in MDA-MB-231 (**B**) and MCF-7 (**E**) cells. The bar graphs show the percentage of MDA-MB-231 cells (**C**) and MCF-7 cells (**F**) in the G_2_/M phases. Cells were stained using PI and subjected to flow cytometric analysis, which included 20 000 events. Data are the mean ± SD of three independent experiments. ^*^P < 0.05, ^**^P < 0.01 and ^***^P < 0.001 by one-way ANOVA.

To explore the different effects of X-ray and carbon ion irradiation on the cell cycle of MDA-MB-231 and MCF-7 human breast cancer cell lines, we assessed the cell cycle distribution of cells 48 h after exposure to X-rays or carbon ions. As shown in Fig. 2, the percentage of cells in the G_2_/M phase markedly increased in a dose-dependent manner compared with that in the untreated control; meanwhile, the percentage of cells in the G_1_ phase and S phase decreased in both cell lines after irradiation with X-rays or carbon ions. In MDA-MB-231 cells, the percentage of cells arrested in the G_2_/M phase was 23.02, 27.3%, 49.02 and 68.44% for X-ray irradiation and 24.97, 51.23, 79.81 and 86.36% for carbon ion irradiation at doses of 0, 2, 4 and 8 Gy, respectively (Figs 2B and C). The proportion of cells in the G_2_*/*M phase was 13.23, 28.52, 56.25 and 77.40% for X-ray irradiation and 14.03, 77.44, 79.01 and 88.87% for carbon ion irradiation in MCF-7 cells (Figs 2E and F). These results illustrate that breast cancer cells were arrested in the G_2_/M phase after irradiation with X-rays or carbon ions, and a more obvious increase in the G_2_/M phase ratio was observed in both cell lines after irradiation with carbon ions compared with X-rays at the same dose (*P* < 0.001 by one-way ANOVA; Figs 2C and D). Moreover, X-ray irradiation induced G_2_/M phase arrest more efficiently in MCF-7 cells than in MDA-MB-231 cells compared with the untreated control, while carbon ions induced G_2_/M arrest effectively in both MDA-MB-231 and MCF-7 cell lines.

### Carbon ion irradiation increased the expression of γ-H2AX more effectively than X-ray irradiation in the human breast cancer cell lines MDA-MB-231 and MCF-7

**Fig. 3.**
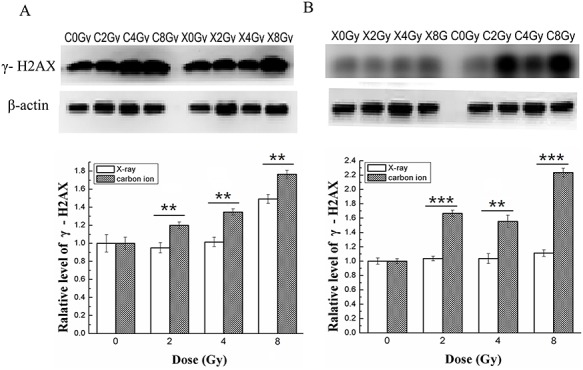
Effects of X-ray or carbon ion radiation on the expression level of γ-H2AX in MDA-MB-231 and MCF-7 cells. The blots show the expression levels of DNA DSB marker γ-H2AX in MDA-MB- 231 (**A**) and MCF-7 (**B**) cells measured by western blotting assay 48 h after X-ray or carbon ion radiation at doses of 0, 2, 4 and 8 Gy. The bar graphs show the relative levels of γ-H2AX in MDA-MB-231 (A) and MCF-7 (B) cells. β-Actin was used as the internal control. Data are the mean ± SD of three independent experiments. ^**^P < 0.01 and ^***^P < 0.001 by one-way ANOVA.

γ-H2AX is a novel biomarker for DNA DSBs. Carbon ion irradiation is known to induce a greater amount of DNA damage, such as DSBs. To further compare the different effects of X-ray and carbon ion irradiation focusing on DNA damage, we evaluated the level of γ-H2AX. In MDA-MB-231 cells, as exhibited in Fig. 3A, the expression level of γ-H2AX increased 1.20-, 1.35- and 1.76-fold after radiation with carbon ions at doses of 2, 4 and 8 Gy, respectively, while these values showed almost no increase after radiation with X-rays at doses of 2 and 4 Gy; the expression level increased 1.0-, 1.01- and 1.49-fold after radiation with X-rays at the same doses compared with the control. In MCF-7 cells, the expression level of γ-H2AX increased 1.66-, 1.55- and 2.24-fold compared with the control after radiation with carbon ions at doses of 2, 4 and 8 Gy, while it increased 1.03-, 1.04- and 1.11-fold after radiation with X-rays at the same doses compared with the control (Fig. 3B). Our data showed that more DSBs were generated by carbon ions, especially in MCF-7 cells. These irreparable DNA DSBs are likely to lead to cell death.

### Carbon ion irradiation induced cell apoptosis more effectively than X-ray irradiation in MDA-MB-231 and MCF-7 human breast cancer cell lines

**Fig. 4.**
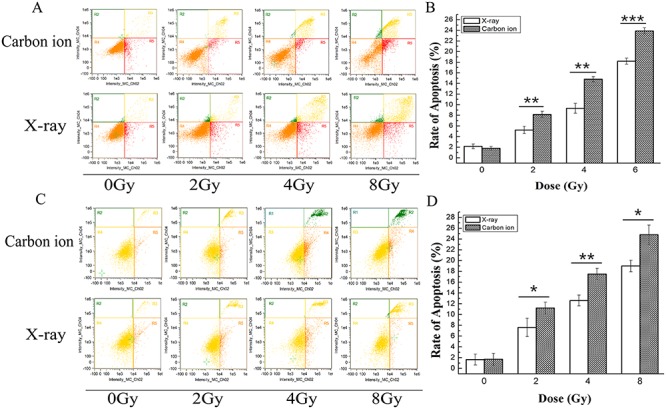
Apoptosis induced by X-rays or carbon ions in MDA-MB-231 and MCF-7 cells determined by flow cytometry 48 h after irradiation. The flow cytometric dot plots show the percentage of specific cell populations (live, early apoptosis and late apoptosis) in MDA-MB-231 (**A**) and MCF-7 (**C**) cells irradiated with X-rays or carbon ions at doses of 0, 2, 4 and 8 Gy. The bar graphs show the percentage of late apoptotic MDA-MB-231 (**B**) and MCF-7 (**D**) cells. Cells were double-stained with annexin V/PI to detect cells undergoing early and late apoptosis, which collected 10 000 events. Data are the mean ± SD of three independent experiments. ^*^P < 0.05, ^**^P < 0.01 and ^***^P < 0.001 by one-way ANOVA.

To examine the different apoptosis-inducing effects of X-ray and carbon ion irradiation in MDA-MB-231 and MCF-7 cell lines, the number of apoptotic cells was quantified using flow cytometric analysis 48 h after treatment, and the results are shown in Fig. 4. Our results showed that the apoptotic cell population increased in a dose-dependent manner in MDA-MB-231 and MCF-7 cells after exposure to X-rays or carbon ions at doses of 0, 2, 4 and 8 Gy. In MDA-MB-231 cells, the percentage of apoptosis/necrosis was 2.16, 5.24, 9.32 and 18.2% for X-ray irradiation, and 1.8, 8.16, 14.8 and 23.9% for carbon ion irradiation (Figs 4A and B). The percentage of apoptotic cells increased 2.4-, 4.3- and 8.4-fold compared with the control after irradiation with X-rays at doses of 2, 4 and 8 Gy, more effectively, it increased 4.5-, 8.2- and 13.3-fold after irradiation with carbon ions. Similarly, the percentage of apoptotic cells was 1.66, 7.6, 12.6 and 19% for X-ray irradiation and 1.7, 11.2, 17.5 and 24.8% for carbon ions irradiation in MCF-7 cells (Figs 4C and D). The percentage of apoptotic cells increased 4.5-, 7.5- and 11.4-fold after irradiation with X-rays and increased 6.6-, 10.3- and 14.6-fold after irradiation with carbon ions at doses of 2, 4 and 8 Gy compared with the control (0 Gy). These results indicated that both X-rays and carbon ions induced cell apoptosis/necrosis in a dose-dependent manner, and carbon ions showed significant superiority over X-rays in killing cells at all doses employed in both cell lines (*P* < 0.05 by one-way ANOVA; Figs 4C and D). Furthermore, it was noticeable that both X-rays and carbon ions induced cell apoptosis more efficiently in MCF-7 cells than in MDA-MB-231 cells.

### Protein expression in MDA-MB-231 and MCF-7 cells after X-ray or carbon ion irradiation

**Fig. 5.**
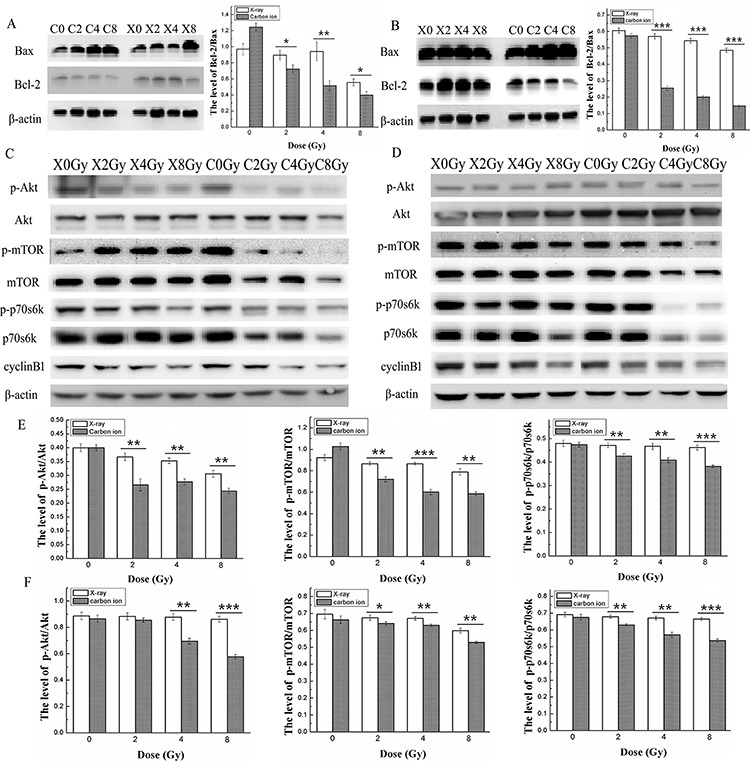
Effects of X-ray or carbon ion radiation on the expression levels of Bax, Bcl-2, the PI3K/Akt/mTOR signalling path way and cyclin B1 in MDA-MB-231 and MCF-7 cells. The blots show the expression levels of the cell apoptosis-related proteins Bax and Bcl-2, the expression levels and phosphorylation level of the Akt/mTOR/p70S6K signalling pathway, and cyclin B1 in MDA-MB-231 (**A**, **C**) and MCF-7 (**B**, **D**) cells measured by western blotting assay 48 h after X-ray or carbon ion radiation at doses of 0, 2, 4 and 8 Gy. The bar graphs show the ratio of p-Akt/Akt, p-mTOR/mTOR and p-p70S6K/p70S6K in MDA-MB-231 (**E**) and MCF-7 (**F**) cells. β-Actin was used as the internal control. Data are the mean ± SD of three independent experiments. ^*^P < 0.05, ^**^P < 0.01 and ^***^P < 0.001 by one-way ANOVA.

To further confirm the different effects of X-ray and carbon ion irradiation-induced cell cycle arrest and apoptosis, the proapoptotic protein Bax, the antiapoptotic protein Bcl-2 and the G_2_/M phase-related protein cyclin B1 were examined using western blot analysis. In MDA-MB-231 cells, as shown in Fig. 5A, compared with the expression level in the control group, the expression level of Bax increased greatly 1.41-, 1.89- and 2.20-fold after exposure to carbon ions at all doses (2, 4 and 8 Gy), while it increased markedly only at 8 Gy X-ray treatment, with a 1.72-fold increase. In contrast to the change in Bax expression level, the expression level of Bcl-2 reduced to 0.95, 0.96 and 0.93 after treatment with X-rays, while it reduced sharply to 0.82, 0.79 and 0.68 compared with the control after treatment with carbon ions at doses of 2, 4 and 8 Gy. Consistent with the expected result, the expression level of cyclin B1 in MDA-MB-231 cells reduced to 0.93, 0.92 and 0.80 after exposure to X-rays at doses of 2, 4 and 8 Gy, and it reduced sharply to 0.92, 0.76 and 0.65 after exposure to carbon ions, respectively. In MCF-7 cells, the expression level of Bax increased 1.33-, 1.52- and 1.59-fold after treatment with carbon ions at doses of 2, 4 and 8 Gy, while it increased weakly 1.13-, 1.15- and 1.21-fold compared with the control at the same doses of X-rays. In contrast to the change in Bax expression level, the expression level of Bcl-2 decreased substantially to 0.78, 0.64 and 0.49 compared with the control after treatment with carbon ions, while a slight reduction was observed at all doses of X-ray. Similar to Bcl-2, the expression level of cyclin B1 reduced sharply to 0.84, 0.83 and 0.72 after exposure to carbon ions at doses of 2, 4 and 8 Gy, while it decreased faintly at the same doses of X-rays (Fig. 5B). Taken together, these results were consistent with the results of enhanced G_2_/M phase arrest and apoptosis and indicated that both human breast cancer cell lines were more sensitive to carbon ion than X-ray irradiation. In particular, in MDA-MB-231 cells, the X-ray irradiation-induced increase in Bax and decreases in Bcl-2 and cyclin B1 were observed only at the dose of 8 Gy, while carbon ions induced significant changes at all doses employed.

To determine whether the activation of the Akt/mTOR/p70S6K signalling pathway, which participates in tumours resistant to radiation therapy [28], was involved in the response of breast cancer cells to carbon ion irradiation, we tested the phosphorylation levels and total expression levels of the Akt/mTOR/p70S6K pathway 48 h after irradiation. In both breast cancer cell lines, the levels of p-Akt, p-mTOR, p-p70S6K, and total Akt, mTOR and p70S6K protein decreased to a larger extent after exposure to carbon ions than after exposure to X-rays (Fig. 5). In MDA-MB-231 cells, the level of p-Akt reduced to ~0.79, 0.72 and 0.57 of that of unirradiated cells after carbon ion irradiation at any dose (2, 4 and 8 Gy), while X-ray irradiation at the same doses caused no significant change in the p-Akt levels. Similar to p-Akt, the expression levels of p-mTOR decreased greatly to 0.52, 0.50 and 0.41, and the expression of p-p70S6K decreased to 0.88, 0.85 and 0.72, respectively, after exposure to carbon ions at all doses employed, while no significant reduction of p-mTOR and p-p70S6K levels was observed after exposure to X-rays at all doses (Figs 5C and E). In MCF-7 cells, the expression levels of p-Akt, p-mTOR and p-p70S6K decreased to a large extent after exposure to carbon ions at doses of 4 and 8 Gy, whereas a significant reduction in p-Akt, p-mTOR, and p-p70S6K levels was observed only at the highest dose (8 Gy) of X-ray irradiation (as shown in Figs 5D and F). These results indicated that carbon ion radiation might inhibit the Akt/mTOR/p70S6K signalling pathway more effectively than X-ray radiation in both breast cancer cell lines. In addition, the X-ray-induced inhibition of the Akt/mTOR/p70S6K pathway was negligible at all doses in MDA-MB-231 cells, while cells treated with 8 Gy X-rays showed a modest inhibition of Akt/mTOR/p70S6K in MCF-7 cells. These results indicated that carbon ion radiation might inhibit activation of the Akt/mTOR/p70S6K signalling pathway more effectively than X-ray radiation in both breast cancer cell lines.

### DISCUSSION

Although radiation therapy is a powerful tool for treating breast cancer, resistance of tumour cells to this treatment remains a serious concern. Breast cancer is still the most frequent cause of cancer-related death in women and ranks second after lung cancer [29]. Recently, heavy-ion radiotherapy, such as carbon ion radiotherapy, has emerged and has become a promising new anticancer strategy; numerous studies have been undertaken to explore the related molecular mechanisms underlying its anticancer effect. Because carbon-ion beams have a well-defined energy distribution and higher relative biological effectiveness, they therefore induce more complex DSB damage and clustered damage around break sites, which is often repaired less efficiently and ultimately induces cell apoptosis more efficiently than traditional X-ray radiotherapy [30–32]. The aim of this study was to compare the different effects of X-ray and carbon ion irradiation on MDA-MB-231 and MCF-7 breast cancer cell lines in terms of cell proliferation, cell colony formation, cell cycle distribution, DNA damage and cell apoptosis and to investigate the role of the Akt/mTOR/p70S6K signalling pathway in radiosensitisation.

Cellular proliferation is regulated primarily by the cell cycle, which consists of four distinct sequential phases (G_0_/G_1_, S, G_2_ and M) [33]. Our study showed that X-ray irradiation induced little inhibition of cell proliferation in both breast cancer cell lines at doses of 2, 4 and 8 Gy, especially at 24 h after irradiation. However, carbon ion irradiation induced obvious inhibition of cell proliferation and colony formation compared with the same dose of X-ray irradiation in both cell lines (Fig. 1). γ-H2AX is a marker for observation of radiation-induced DSBs because the phosphorylation of histone H2AX on Ser-139 occurs rapidly after irradiation, and dephosphorylation occurs after the repair of DNA [34]. γ-H2AX acts as a sensor and recruits other proteins involved in the DNA damage response, which regulate cell cycle progression, before or during DNA replication (G_1_/S and intra-S checkpoints) and before cell division (G_2_/M checkpoint) [35]. The results obtained in this study showed that the expression level of γ-H2AX was obviously elevated in a dose-dependent manner after carbon ion treatment at all doses employed, while a significant effect was observed only at the highest dose (8 Gy) after radiation with X-rays in MDA-MB-231 cells (Fig. 3A). Similar to MDA-MB-231, the expression level of γ-H2AX in MCF-7 cells increased more significantly after carbon ion treatment than X-rays at all doses employed (Fig. 3B). These results indicate that carbon ion irradiation induced DSB more effectively than X-ray irradiation at the same dose in both breast cancer cell lines. It is widely known that cells are blocked in the G_2_/M phase during DNA damage, and cells are more sensitive to radiotherapy in the G_2_/M phase [36]. The accumulation of cells in the G_2_/M phase during arrest allows cell death, and it may be an efficient strategy in cancer therapeutics [37]. In the present study, cell cycle analyses of MDA-MB-231 and MCF-7 cells by flow cytometry showed that carbon ions inhibited cell cycle progression (G_2_/M arrest) more effectively than X-rays (Fig. 2) at the same dose. We further explored the different effects of X-rays and carbon ions on a key regulator of cell cycle, cyclin B1, and observed a significant decrease in the expression level of cyclin B1 in a dose-dependent manner in MDA-MB-231 and MCF-7 cells after treatment with carbon ions (Fig. 5), which was consistent with G_2_/M cell cycle arrest. These results are consistent with a report by Keta *et al*. [38], which showed that carbon ion irradiation induced a high expression level of γ-H2AX and a high percentage of cells in the G_2_/M phase in non-small-cell lung cancer cells and indicated that more DSBs are produced in response to carbon ions irradiation in tumour cells. If potential DNA damage is not adequately repaired, regulated cell death (apoptosis/necrosis) will be induced. Tumour cells can acquire resistance to apoptosis by downregulating proapoptotic proteins such as Bax or by upregulating antiapoptotic proteins such as Bcl-2 [29]. Our results in this study demonstrate that carbon ion irradiation showed significant superiority over X-ray irradiation in inducing cell apoptosis/necrosis for both breast cancer cell lines at all doses used (Fig. 4). Moreover, we noted a dose-dependent increase in Bax level and a dose-dependent decrease in Bcl-2 level after irradiation with carbon ions at all doses in both MDA-MB-231 and MCF-7 cell lines, which changed obviously only at the highest dose (8 Gy) after irradiation with X-rays in MDA-MB-231 cells (Fig. 5). Taken together, these results indicate that carbon ion irradiation showed marked superiority in inhibiting cell proliferation and colony formation, inducing G_2_/M arrest, accumulating DNA damage and apoptosis/necrosis in both breast cancer cell lines compared with X-rays. Furthermore, we found that MDA-MB-231 cells were more resistant to X-ray radiation than the MCF-7 cell line, and they were both sensitive to carbon ion radiation. Our results coincide with those of another study that indicated that MDA-MB-231 cells were more radioresistant than MCF-7 cells [5].

The serine/threonine protein kinase Akt regulates cell survival, growth and proliferation through phosphorylation of different downstream substrates, such as mTOR. Additional phosphorylation of Akt induced by X-ray irradiation is responsible for the repair of radiation-induced DNA damage [39, 40]. mTOR plays a central role in cell growth regulation by integrating signals from growth factors, nutrients and cellular energy levels, and mTOR phosphorylation promotes cell growth and cell cycle progression via regulation of various signalling pathways, including the PI3K/Akt/mTOR pathway [22, 39, 40]. As a downstream target of mTOR, p70S6K is usually phosphorylated to promote cell proliferation and survival, and suppressing the activity of p70S6K is predicted to inhibit cell cycle regulatory proteins [25, 41]. Recent reports have demonstrated that the PI3K/Akt/mTOR pathway is frequently altered in human breast cancer, and both genetic and biochemical data suggest that aberrant activation of the PI3K/Akt/mTOR pathway contributes to breast cancer development and tumourigenesis [42]. Moreover, the PI3K/Akt/mTOR signalling pathway has been extensively confirmed to be critical for radiotherapy resistance in various cancer types [43, 44]. In addition, some small molecular drugs targeting individual PI3K, Akt or mTOR signalling proteins or dual blockade of PI3K and mTOR have been reported to enhance radiosensitivity in prostate cancer, oral squamous cell carcinoma and lung cancer [21, 45, 46]. To explore the mechanisms by which two breast cancer cell lines are more sensitive to carbon ions, we detected the activation level and the total expression level of the Akt/mTOR/p70S6K pathway. According to the western blot results shown in Fig. 5, we observed that inhibition of the Akt/mTOR/p70S6K pathway by carbon ion irradiation was obvious in both MDA-MB-231 and MCF-7 breast cancer cells, while that of X-rays was negligible. Furthermore, both the phosphorylation level and the total expression level of the Akt/mTOR/p70S6K pathway decreased greatly after exposure to carbon ions at all doses employed compared with those after X-ray exposure in MDA-MB-231 cells (Figs 5C and E), which has been proven to be related to the radiation sensitivity of carbon ions in SHG44 and HeLa cells and a mouse squamous carcinoma cell line [14, 25]. In MCF-7 cells, carbon ions induced slight inhibition of the Akt/mTOR/p70S6K pathway at the lower dose (2 Gy), while their effect was significant and obvious at higher doses (4 and 8 Gy) compared with that of X-rays (Figs 5D and F). Although some research certified that carbon ion irradiation inhibited the phosphorylation level but not the total expression level of proteins of the Akt/mTOR/p70S6K pathway [14, 25], our results support recent research that indicated that high-LET radiation decreases both the phosphorylation level and the total expression level of the Akt/mTOR/p70S6K pathway proteins [27].

## CONCLUSIONS

In summary, the present study showed that carbon ion irradiation suppressed cell proliferation and colony formation and induced G_2_/M arrest, DSB formation and apoptosis/necrosis more effectively in both breast cancer cell lines than X-ray irradiation. In addition, the inhibition effect of the Akt/mTOR/p70S6K pathway by carbon ions was superior to that of X-rays at the same dose, indicating that the prominent superiority of carbon ions over X-rays in killing both breast cancer cell lines might result from the inhibition of the Akt/mTOR/p70S6K pathway. These results indicated that carbon ions could be chosen to target cancer stem cells, especially X-ray radiation-resistant cancer cells such as MDA-MB-231 cells.

## CONFLICT OF INTEREST

None declared.
